# Analysis of the *NUDT15* gene and metabolites of azathioprine in Japanese patients with inflammatory bowel disease

**DOI:** 10.1186/s12876-023-02881-6

**Published:** 2023-07-15

**Authors:** Shizuka Suzuki, Kan Uchiyama, Yutaro Motoi, Yuuki Yoshii, Yukari Inoue, Takahiro Kubota, Shunichi Odahara, Yuichiro Ohtaki, Shinichiro Takami, Zensho Ito, Nobuhiro Sato, Toshifumi Ohkusa, Shigeo Koido, Masayuki Saruta

**Affiliations:** 1 Division of Gastroenterology and Hepatology, Department of Internal Medicine, The Jikei University Kashiwa Hospital, 163-1 Kashiwa, Kashiwa-Shi, Chiba 277-8567 Japan; 2 Department of Biopharmaceutics, Faculty of Pharmaceutical Sciences, Niigata University of Pharmacy and Applied Life Sciences, F103a, 265-1 Higashijima, Akiha-Ku, Niigata City, 956-8603 Japan; 3 Department of Microbiota Research, Juntendo University Graduate School of Medicine, 3-3-1 Hongo, Ochanomizu KS Building 4F 405 Bunkyo-Ku, Tokyo, 113-0033 Japan; 4 Division of Gastroenterology and Hepatology, Department of Internal Medicine, The Jikei University School of Medicine, 3-19-18 Nishishinbashi, Minato-Ku, Tokyo, 105-0003 Japan

**Keywords:** NUDT15, ITPA, Azathioprine, IBD

## Abstract

**Background:**

Thiopurines continue to play an important role in the treatment of inflammatory bowel disease (IBD). It is well known that thiopurines can cause several adverse reactions. Especially, hematopoietic toxicity may lead to severe agranulocytosis. In a previous prospective study, we investigated the relationship between inosine triphosphate pyrophosphatase (*ITPA*) c.94c > a polymorphism, 6-thioguanine nucleotide (6-TGN) concentration and toxicity.

**Methods:**

To clarify the cause of thiopurine toxicity, we analysed nucleoside disphosphate-linked moiety X-type motif 15 (*NUDT15*) gene polymorphisms, i.e., R139C, V18I, and V19_V19insGV, and measured 6-mercaptopurines and 6-methylmercaptopurines (6-MMP) using the archived blood samples collected from 49 IBD patients for our previous study.

**Results:**

The *ITPA* c.94c > a polymorphism was detected in 19 patients (38.7%, all heterozygous). The R139C polymorphism was found in 10 patients (20.4%, 1 homozygous, 9 heterozygous), V18_V19insGV in 7 patients (14.3%, all heterozygous), and V18I in 2 patients (4.08%, all heterozygous). Although R139C was more strongly associated with leukopenia than c.94c > a, there were no significant correlations with 6-TGN and 6-MMP levels, as for c.94c > a. The leukopenia incidence rates for each gene polymorphism were 0% in those with all wild-type genes, 21.4% for c.94c > a only, 42.9% for *NUDT15* polymorphism (s) only, and 80.0% for both polymorphisms.

**Conclusions:**

All cases of leukopenia were associated with *ITPA* c.94c > a and/or polymorphism of *NUDT15* and the risk of developing leukopenia was synergistically increased by *ITPA* and *NUDT15* gene polymorphism. However, there was no association between the level of azathioprine metabolites and these polymorphisms.

## Background

Inflammatory bowel disease (IBD) involves chronic inflammation of the intestinal tract of unknown cause. Although many treatments to induce remission of IBD have been developed, thiopurines continue to play an important role in the treatment of IBD. It is well known that thiopurines can cause several adverse reactions (ARs), such as leukopenia, alopecia, hepatitis, and pancreatitis. In particular, hematopoietic toxicity may lead to severe agranulocytosis.

**Fig. 1:**
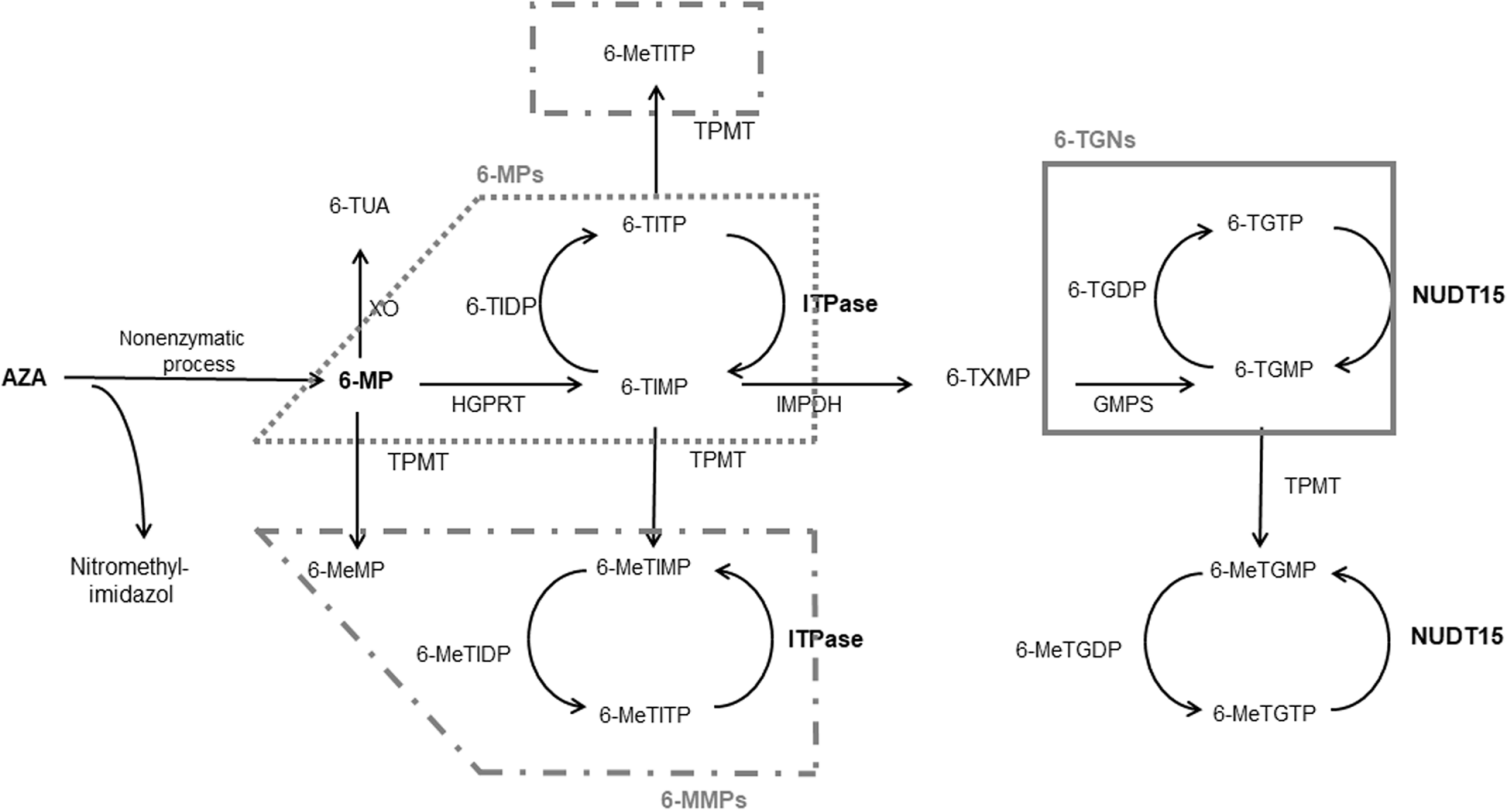
Metabolism and transportation of AZA/6MP and its metabolites. XO, xanthine oxidase; TPMT, thiopurine *S*-methyltransferase; HGPRT, hypoxanthine guanine phosphoribosyl transferase; ITPase, inosine triphosphate pyrophosphatase; IMPDH, inosine monophosphated dehydrogenase; GMPS, guanosine monophosphate synthetase; NUDT15, nucleoside diphosphate-linked moiety X-type motif 15; AZA, azathioprine, 6-MP, 6-mercaptopurine; 6-TUA, 6-thiouric acid; 6-MeMP, 6-methylmercaptopurine; 6-TIMP, 6-thioinosine monophosphate; 6-TIDP, 6-thioinosine diphosphate; 6-TITP, 6-thioinosine triphosphate; 6-MeTIMP, 6-methylthionosine monophosphate; 6-MeTIDP, 6-methylthionosine diphosphate; 6-MeTITP, 6-methylthionosine triphosphate; 6-TXMP, 6-thixanthosine 5’-monophosphate; 6-TGMP, 6-thioguanosine monophosphate; 6-TGDP, 6-thioguanosine diphosphate; 6-TGTP, 6-thioguanosine triphosphate; 6-MeTGMP,; 6-MeTGMP, 6-methylthioguanine monophosphate; 6-MeTGDP, 6-methylthioguanine diphosphate; 6-MeTGTP, 6-methylthioguanine triphosphate; 6-MMPs, 6-methylmercaptopurines; 6-MPs, 6-mercaptopurines; 6-TGNs, 6-thiguanine nucleotide

Azathioprine (AZA) is a prodrug of 6-mercaptopurine (6-MP) that is converted to 6-MP in a nonenzymatic pathway in erythrocytes (Fig. 1). 6-MP is then converted to various metabolites by three metabolic enzymes: xanthine oxidase (XO), thiopurine *S*-methyltransferase (TPMT), and hypoxanthine guanine phosphoribosyl transferase (HGPRT). The activity of TPMT influences the content of 6-thioguanine nucleotide (6-TGN), which increases when activity of the enzyme is reduced due to polymorphism of the gene. Inosine triphosphate pyrophosphatase (ITPase) converts 6-thioinosine triphosphate (6-TITP) to 6-thioinosine monophosphate (6-TIMP), and decreased ITPase activity is thought to lead to accumulation of 6-TITP. In our previous retrospective study, we reported that polymorphism of *ITPA* is closely related to ARs of thiopurines, especially hematopoietic toxicity, in Japanese patients with IBD [1]. A recent Korean study has revealed that nucleoside diphosphate-linked moiety X-type motif 15 (*NUDT15*) polymorphism is associated with ARs, in particular, bone marrow toxicity and alopecia [2]. NUDT15, which belongs to the nudix hydrolase enzyme family, converts the thiopurine active metabolites 6-thioguanosine triphosphate (6-TGTP) and 6-thiodeoxyguanosine triphosphate (6-TdGTP) to 6-thioguanosine monophosphate (6-TGMP) and 6-thiodeoxyguanosine monophosphate (6-TdGMP), respectively. Carriers of polymorphism in this gene have lower enzyme activity, and it has been reported that individuals who are homozygous for polymorphism almost certainly develop bone marrow toxicity [3].

Nevertheless, *NUDT15* polymorphism alone is not sufficient to explain all thiopurine intolerance events, including bone marrow toxicity [4]. Although a higher 6-TGN level is thought to be one of the causes of leukopenia, it has been reported that *NUDT15* polymorphism is not related to the content of 6-TGNs [5]. Similarly, our previous prospective study monitoring 6-TGN levels revealed that *ITPA* polymorphism is associated with the development of myelotoxicity, with no significant difference in 6-TGN level between groups with and without ARs [6]. There was a background that the NUDT15 gene polymorphism had not been reported at the time of our previous prospective study. Therefore, since the relationship between NUDT15 gene polymorphism and thiopurine toxicity has been reported, it is important to analyze the content of intermediate metabolites other than 6-TGN to elucidate the cause of thiopurine toxicity. In this study, we additionally analyzed the *NUDT15* gene and measured intermediate metabolites of AZA, namely, 6-mercaptopurines (6-MPs) and 6-methylmercaptopurines (6-MMPs), using archived blood samples from a previous study. We also investigated relationships between AZA metabolites and polymorphism(s) in various genes involved in AZA metabolism, i.e., *TPMT*, *NUDT15* and *ITPA*.

## Methods

### Subjects

In this study, we analyzed intermediate metabolites levels and *NUDT15* polymorphisms in IBD patients using archived samples from previous research [6]. From March 2008 to June 2011, 49 Japanese IBD patients who received AZA for 52 weeks were enrolled. In Western countries, an AZA dosage of 2–3 mg/kg/day is recommended for the treatment of IBD patients [7]; however, lower dosages (0.6–1.2 mg/kg/day) are used in Japanese individuals because of their relatively heightened sensitivities [8]. In this previous study, the initial AZA dosage was 1.0 mg/kg/day, and whole blood samples were drawn at 0, 1 and 2 weeks and every 4 weeks thereafter until week 52 or the onset of adverse reactions. Blood samples were generally collected in the morning before administration of thiopurine. These blood samples were stored in a -30 °C freezer after the previous study was completed. In our previous study, 48 of 50 patients were observed; we excluded two patients due to cancellation of follow-up because one patient moved and the other complained of hair loss. In the latter case, hair loss occurred immediately after starting the drug, and the patient stopped taking AZA at his own discretion; the degree of hair loss was so mild that it was difficult to diagnose alopecia due to thiopurine. However, improvement in hair volume was objectively confirmed in follow-up. In the present study, we analyzed the archived blood samples of forty-nine patients, including the patient who experienced hair loss. The mean age was 33.8 (range 16–70), and 30 males (61.2%) and 19 females (38.7%) were included. Thirty patients had ulcerative colitis (UC) (61.2%), and 19 had Crohn’s disease (CD) (38.8%).

### TPMT, ITPA, NUDT15

For *TPMT* gene analysis, mutant alleles that reportedly reduce enzyme activity, i.e., *3C and *6, were analyzed by PCR–RFLP; other polymorphism were examined by sequence analysis. For *ITPA* gene analysis, we screened for the c.94c > a polymorphism, which causes reduced ITPase activity [1, 9]. For *NUDT15* gene analysis, we evaluated the R139C, V18I, and V18_V19insGV polymorphism, which cause reduced enzyme activity [10, 11], by PCR amplification and Sanger sequencing. The primer sequences used were based on those reported by Moriyama et al., and those for exon 1 were modified (forward, 5’-GTG GTG CCG AGG TTG GTA AG-3’ and reverse, 5’-ACC TCA CAG ACG AAC TCC CA-3’).

### 6-TGNs, 6-MMPs, 6-MPs

6-TGN levels in red blood cells (RBCs) were measured using high-performance liquid chromatography (HPLC), as described in our previous study [6, 12, 13]. Liquid chromatography tandem mass spectrometry (LC–MS/MS) was used to assess 6-MMPs and 6-MPs [14]. The average concentration of various metabolites was calculated from the 8th to the 52nd week after the start of administration.

### Definition of ARs

In this study, leukopenia was defined as a white blood cell count of 2500/μL or less. Hepatotoxicity was defined as elevated serum alanine aminotransferase and aspartate aminotransferase levels above the upper limit of the normal range.

### Statistical analysis

All statistical analyses were performed using IBM SPSS Statistics version 22. The chi-square test was used for cross-tabulation, but Fisher's exact test was applied when cells with less than the minimum expected frequency were detected. The incidence of ARs by each gene was compared with the chi-square test. Comparisons of various averages between groups were examined by the *t*-test or ANOVA. Logistic regression analyses were employed to evaluated the association between ARs or leukopenia and various risk factors. Statistical significance was declared at the 0.05 level. The chi-square test was used to compare the obtained genotype distribution with the results of previous reports in Japan [15, 16].

### Ethical considerations

These experiments were approved by the Ethics Committee of the Jikei University School of Medicine (27–323(8208)) and Niigata University of Pharmacy and Applied Life Sciences (H29-001). For the previous study (19–190 (5121)), we explained the purpose of the study and the methods involved to all participants prior to enrollment, and each individual provided written informed consent.

In conducting additional analysis with an existing sample, we announced the medical research on the bulletin board in front of the outpatient consultation room and asked whether participants wished to be excluded from the study.

## Results

### TPMT, ITPA, NUDT15

All patients carried the wild-type *TPMT* gene sequence. The *ITPA* c.94c > a polymorphism was detected in 19 patients (38.7%, all heterozygous). With respect to the *NUDT15* gene, the R139C polymorphism in exon 3 was detected in 10 patients (20.4%, 1 homozygous, 9 heterozygous; allele frequency 0.112); the V18_V19insGV polymorphism in exon 1 was found in 7 patients (14.3%, all heterozygous; allele frequency 0.0714) and the V18I polymorphism in exon 1 in 2 (4.08%, all heterozygous; allele frequency 0.0204). Six of the 7 patients harboring the V18_V19insGV polymorphism also carried the R139C polymorphism, and one showed R139C homozygosity. Wild-type genes were detected in 22 of the 49 patients (44.9%). Fourteen patients (28.6%) carried only the *ITPA* c.94c > a polymorphism and 8 patients (16.3%) only *NUDT15* polymorphism(s); 5 patients (10.2%) carried both *ITPA* c.94c > a and *NUDT15* polymorphisms. There was no significant difference in the allele frequency of each polymorphism compared to previous reports [15, 16] in Japanese (*ITPA* c.94c > a: *p* = 0.121, *NUDT15*: *p* = 0.400).

### ARs

ARs occurred in 15 of the 49 patients (30.6%), with some overlap, including leukopenia in 10 (20.4%), alopecia in 4 (8.2%), agranulocytosis in 1 (2.0%), hepatotoxicity in 1 (2.0%), and eruption in 1 (2.0%) (Table1). These patients consisted of 5 males (33.3%) and 10 females (66.7%). Onset was reported to occur from 7 to 364 days after AZA administration. Seven patients discontinued AZA therapy due to ARs.

**Table 1:** List of patients with adverse reactions

NO	Age	Gender	Disease	Adverase reactions	*TPMT*	*ITPA*	*NUDT15*			Duration	Continuation
					*3C	c.94c > a	R139C	V18l	V18_V19insGV	(Days)	of treatent
1	38	F	UC	Leukopenia	-	±	-	±	-	116	Temporary withdrawal
2	55	M	UC	Leukopenia	-	±	-	-	-	117	Dosage reduction
3	24	M	CD	Leukopenia	-	±	-	-	-	7	Continuation
4	66	F	UC	Leukopenia	-	-	±	-	-	40	Discontinuation
5	58	M	UC	Leukopenia	-	±	±	-	-	15	Discontinuation
6	37	F	UC	Leukopenia	-	-	-	-	±	135	Continuation
7	23	F	CD	Leukopenia	-	-	±	-	±	81	Temporary withdrawal
8	22	F	CD	Leukopenia	-	±	±	-	-	91	Discontinuation
9	48	F	UC	Leukopenia	-	±	-	-	-	364	Dosage reduction
10	16	F	UC	Agranulocytosis, Alopecia	-	±	+ / +	-	±	21	Discontinuation
11	31	F	UC	Alopecia	-	±	-	-	-	59	Dosage reduction
12	18	M	UC	Alopecia	-	-	±	-	±	36	Discontinuation
13	50	F	UC	Alopecia	-	-	-	-	-	21	Discontinuation
14	65	F	UC	Hepatotoxicity	-	-	-	-	-	60	Discontinuation
15	24	M	CD	Eruption	-	-	-	±	-	147	Temporary withdrawal

*AZA* azathioprine, *IBD* inflammatory bowel disease, *F* female, *M* male, *UC* ulcerative colitis, *CD* Crohn's disease, *TMPT* thiopurine S-methyltransferase, *ITPA* inosine triphosphate pyrophosphatase, *NUDT15* nucleoside disphosphate-linked moiety X-type motif 15

*indicates the type of gene mutation

**Fig. 2:**
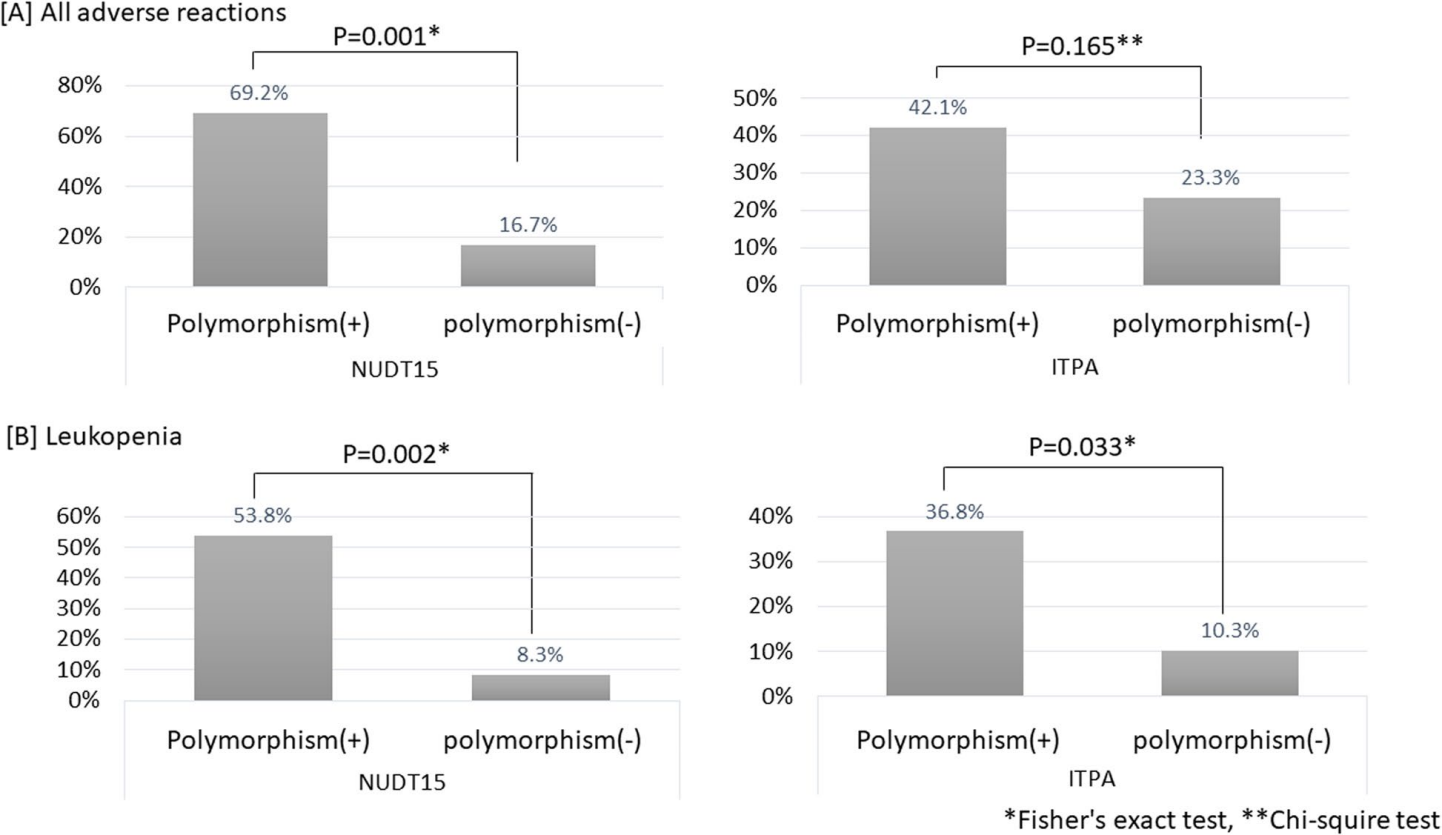
Frequency of adverse reactions by gene polymorphism pattern. The incidence of leukopenia was highest in the order of wild type, only 94 c > a, only polymorphism(s) of *NUDT15*, both polymorphisms. NUDT15, nucleoside diphosphate-linked moiety X-type motif 15; ITPA, inosine triphosphate pyrophosphatase

**Fig. 3:**
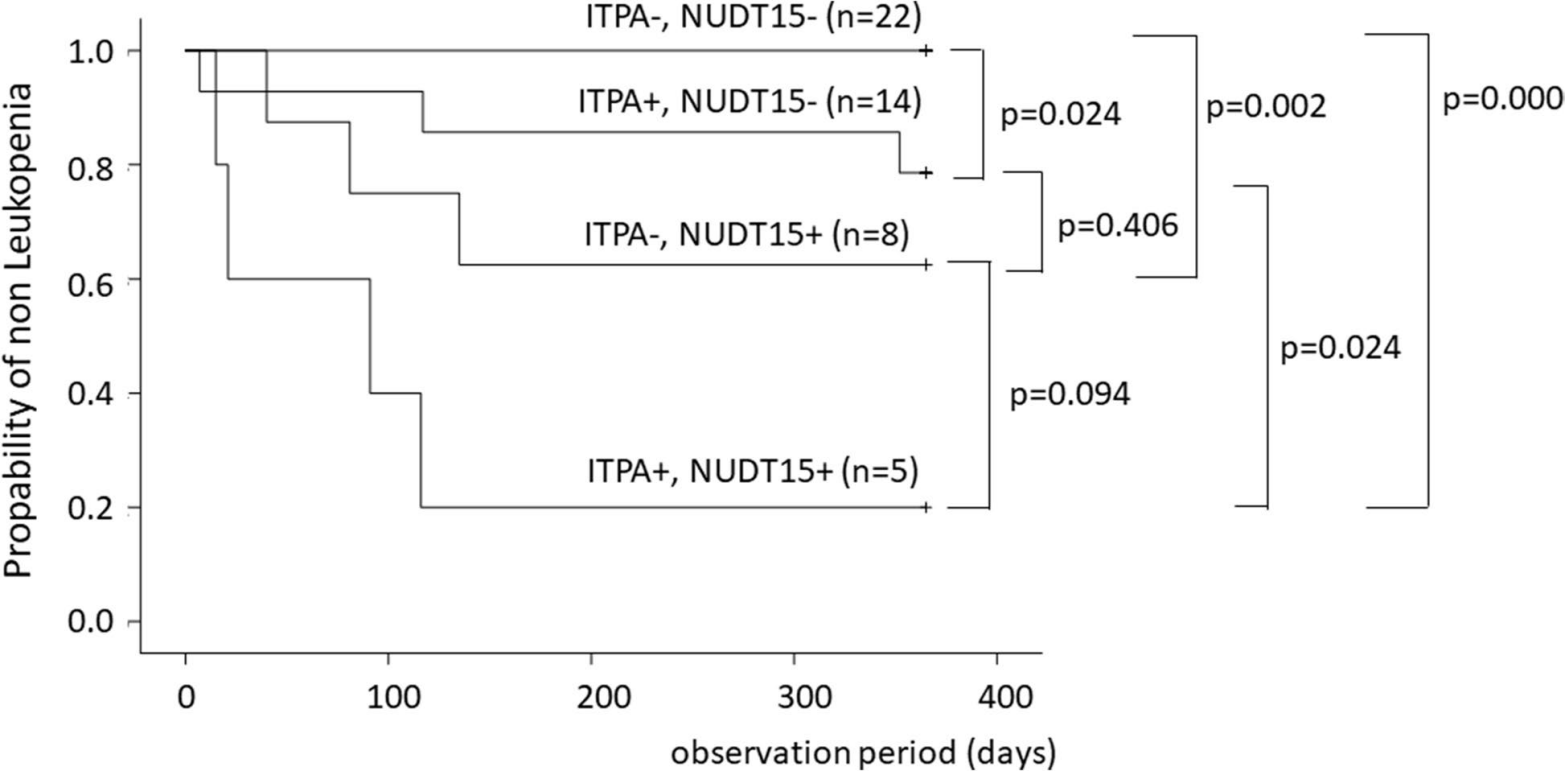
Estimates of cumulative incidence of leukopenia by each gene polymorphism pattern. It was confirmed that the number of days until leukopenia developed tended to decrease in the order of both polymorphisms, only polymorphism(s) of *NUDT15*, and only *ITPA*, but there was no statistically significant difference. ITPA, inosine triphosphate pyrophosphatase; NUDT15, nucleoside diphosphate-linked moiety X-type motif 15

The incidence of all ARs tended to be higher in patients with than in those without the *ITPA* c.94c > a polymorphism, but there was no significant difference (42.1% vs. 23.3%, *P* = 0.165). In addition, the incidence of all ARs was significantly higher in patients with *NUDT15* (*P* = 0.001) than in patients without (69.2% vs. 16.7%, *P* = 0.001) *NUDT15* gene polymorphism. With respect to leukopenia, the probability was higher in patients with than that in those without the c.94c > a polymorphism (36.8% vs. 10.3%, *P* = 0.033), as we previously reported. Similar to previous studies, the incidence of leukopenia in patients with *NUDT15* polymorphisms was higher than that in those without *NUDT15* polymorphisms (53.8% vs 8.3%, *P* = 0.002). Furthermore, the incidence of leukopenia occurred in the following order: wild-type, c.94c > a only, *NUDT15* polymorphism(s) only, and both polymorphisms (0%, 21.4%, 37.5%, and 80.0%, respectively) (Fig. 2). We also confirmed that the number of days until leukopenia developed tended to decrease along the order of both polymorphisms, *NUDT15* polymorphism(s) only, and c.94c > a only, but without a significant difference (60.8 ± 50.5, 85.3 ± 47.6, and 158.7 ± 176.234 days, respectively). The incidence of cumulative nonleukopenia based on the various genotypes was higher in the order of wild-type, c.94c > a only, *NUDT15* polymorphism(s) only, and both polymorphisms, with significant differences (Fig. 3).

The only patient who harbored a homozygous R139C polymorphism developed severe alopecia and agranulocytosis, and this patient also carried the c.94c > a polymorphism.

**Fig. 4:**
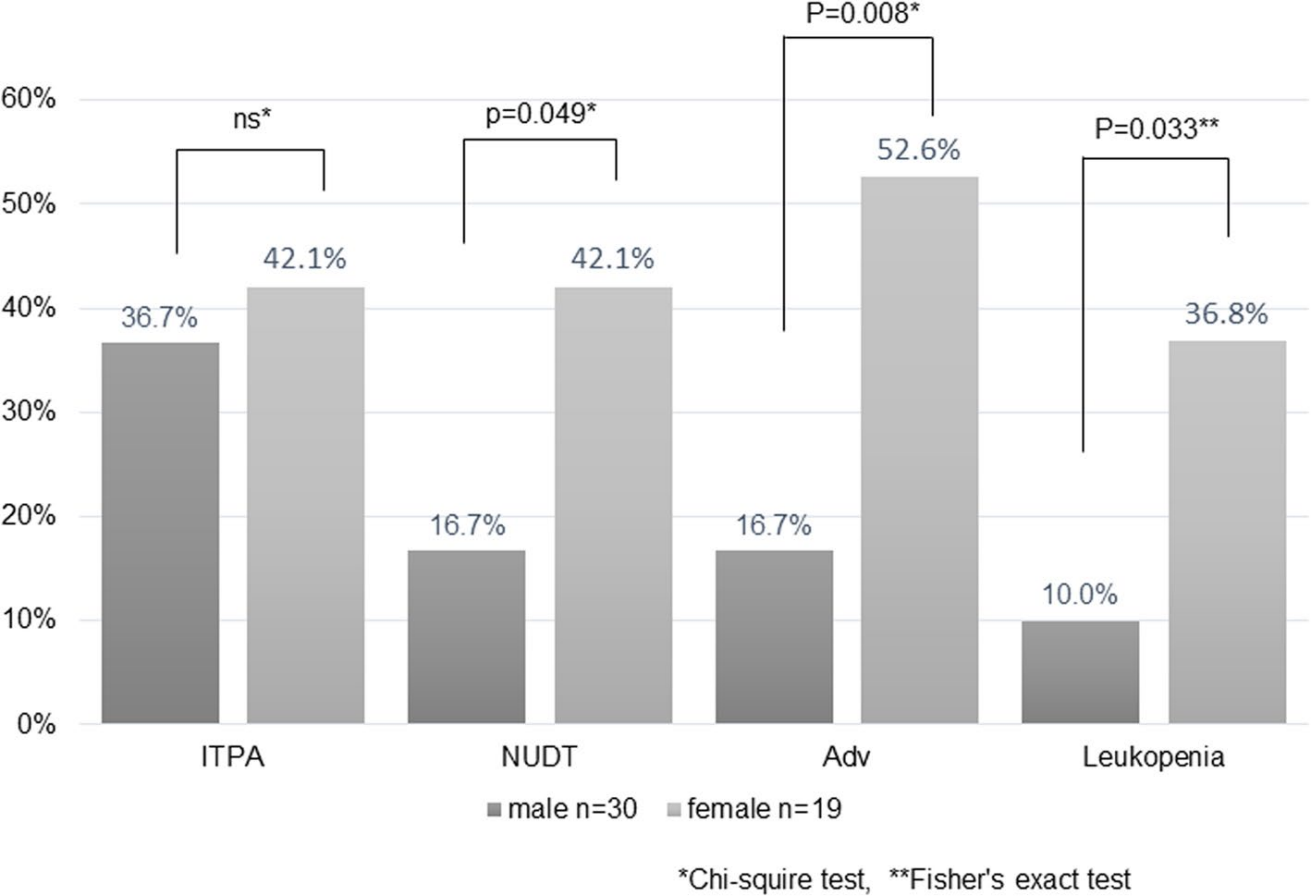
Gender differences in genetic polymorphisms and adverse events. The percentage of female in patients with adverse reactions was higher than that in patients without leukopenia. However, the polymorphism prevalence of the *NUDT15* gene was significantly higher in female. ITPA, inosine triphosphate pyrophosphatase; NUDT15, nucleoside disphosphate-linked moiety X-type motif 15; Adv, Adverse event

Similar to our previous report, the percentage of females among patients with ARs was higher than that among patients without ARs (66.7% vs. 26.5%, *P* = 0.010), and the proportion of females among patients with leukopenia was higher than that among patients without leukopenia (70.0% vs. 30.8%, *P* = 0.029) (Fig. 4). Moreover, the prevalence of *NUDT15* gene polymorphism was significantly higher in females and males (*P* = 0.049), whereas the prevalence of c.94c > a was not significantly different (*P* = 0.466).

Multivariate analysis was performed using ARs as the dependent variable and female sex, *ITPA* c.94c > a and *NUDT15* polymorphism(s) as explanatory variables, and the results showed that *NUDT15* polymorphism(s) was an independent factor [OR = 11.1; 95%CI: 2.12–58.1]. When multivariate analysis was performed with leukopenia as the dependent variable, c.94c > a and *NUDT15* polymorphism(s) were independent factors [c.94c > a: OR = 16.9; 95%CI: 1.45–197.6, *NUDT15* polymorphism(s): OR = 25.5; 95%CI: 2.37–275.0].

Although the number of discontinuation of AZA cases was 3 of 19 (15.8%) among *ITPA* c.94c > a carriers and 8 of 13 (61.5%) among *NUDT15* polymorphism(s) carriers, for the former, all patients who required discontinuation also carried a polymorphism in *NUDT15*; no carriers of *ITPA* polymorphism alone required discontinuation. Overall, those who harbored the *ITPA* polymorphism alone who developed ARs continued taking thiopurines with temporary withdrawal or dosage reduction (Table 1).

**Fig. 5:**
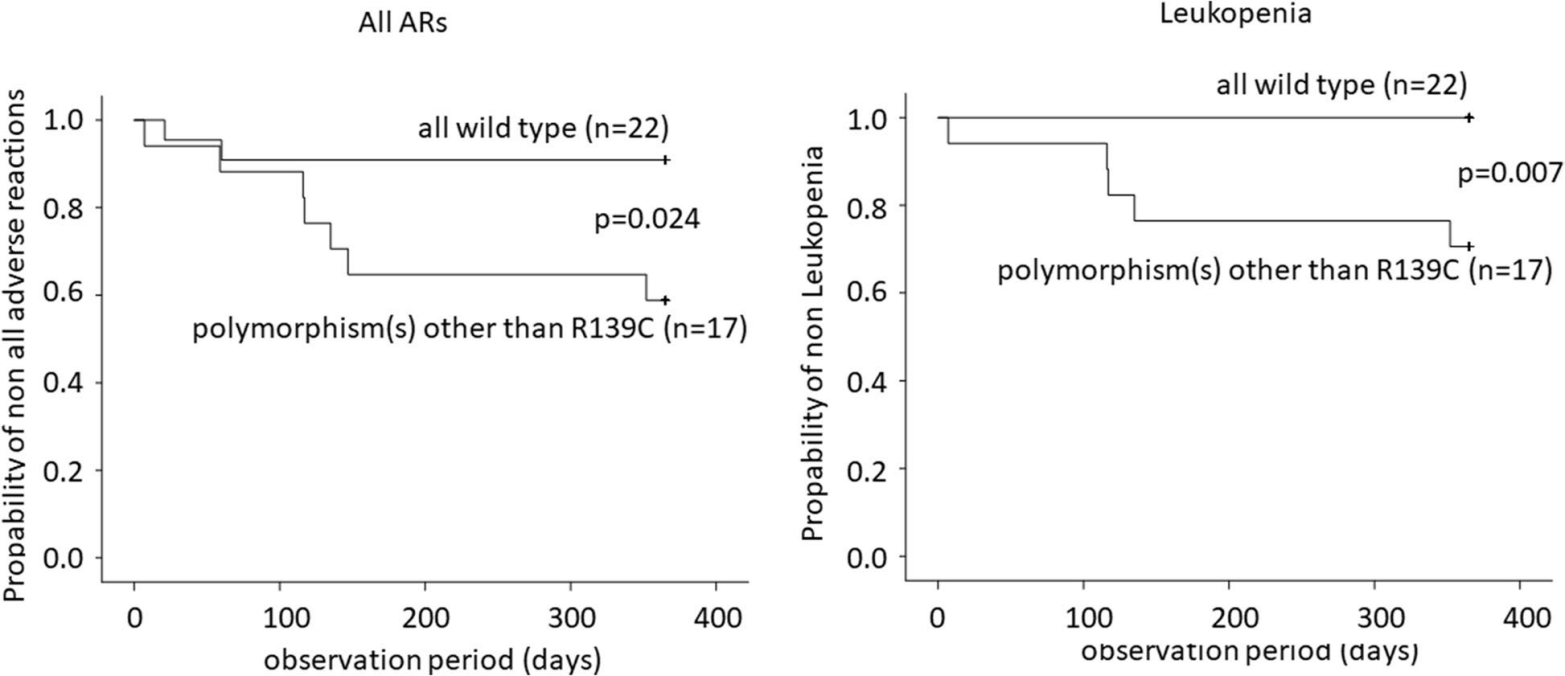
Estimates of cumulative incidence: All wild-type vs. polymorphisms other than R139C. Patients with polymorphisms other than R139C had a significantly higher incidence of all adverse reactions and leukopenia than all wild-type patients

Seventeen of the 49 (34.7%) patients did not have the R139C polymorphism but did carry other polymorphisms. All of these patients had a significantly higher incidence of all ARs and leukopenia than those who carried wild-type genes (41.2% vs. 9.1%; *p* = 0.026, 29.4% vs. 0%; *P* = 0.011). Survival analysis using the Kaplan–Meier and log rank tests also confirmed significant differences in all ARs and leukopenia (*P* = 0.024, *P* = 0.007) (Fig. 5).

### 6-TGN, 6-MP, and 6-MMP concentrations

The average content of 6-TGN in patients with the c.94c > a polymorphism was generally higher than that in patients carrying wild-type genes, though there was no significant difference between the groups. Despite a strong association between *NUDT15* polymorphism(s) and leukopenia, the average 6-TGN level in *NUDT15* polymorphism carriers tended to be consistently lower than that of patients with wild-type genes.

No significant correlation between the average 6-MMP concentration and c.94c > a or *NUDT15* polymorphism was observed. In addition, 6-MPs were often undetectable, probably because this intermediate is rapidly metabolized by XO, TPMT, and HGPRT and thus unstable. Hene, the average level of 6-MPs was very low compared to that of 6-TGNs and 6-MMPs. Similar to 6-TGNs and 6-MMPs, the average concentration of 6MPs did not differ significantly with genotype (Table 2).

**Table 2:** Average concentration of intermediate metabolites by gene polymorphism pattern

Gene polymorphism pattern	Type1 (*n* = 22)	Type2 (*n* = 14)	Type3 (*n* = 8)	Type4 (*n* = 5)	
	*ITPA-/NUDT15-*	*ITPA* + */NUDT15-*	*ITPA-/NUDT15* +	*ITPA* + */NUDT15* +	*P* value
6-TGNs (pmol/8 × 10^8^RBCs)	360.9 ± 158.1	448.4 ± 204.1	286.6 ± 149.4	436.3 ± 146.4	0.201
6-MMPs (pmol/8 × 108RBCs)	321.5 ± 141.0	249.4 ± 110.6	252.3 ± 179.8	518.0 ± 526.1	0.091
6-MPs (pmol/8 × 108RBCs)	6.82 ± 7.47	10.4 ± 12.4	5.53 ± 6.65	4.13 ± 3.62	0.482
					ANOVA

*ITPA* inosine triphosphate pyrophosphatase, *NUDT15* nucleoside disphosphate-linked moiety X-type motif 15, *6-TGNs* 6-thiguanine nucleotide, *6-MMPs* 6-methylmercaptopurines, *6-MPs* 6-mercaptopurines

Although a 6-MMP content above 5,700 pmol/8 × 10^8^ RBCs is considered to be related to hepatotoxicity [17], the 6-MMP level in patients with hepatotoxicity was rather low (864 pmol/8 × 10^8^ RBCs at most). In general, patients with higher 6-MMP contents did not exhibit hepatotoxicity (1,640 pmol/8 × 10^8^ RBCs at most).

## Discussion

The mechanism by which ARs occur due to thiopurines has remained unclear for many years. In Asians, *TPMT* polymorphism is not a sufficient factor for myelosuppression. Although several studies have reported that *ITPA* polymorphism can cause thiopurine-induced myelosuppression, only 36.8% of c.94c > a polymorphism carriers in our previous prospective study exhibited leukopenia [6]. In other words, the *ITPA* c.94c > a polymorphism has only been demonstrated to be one of several causes. Additionally, a research group in Korea reported on a global discovery, i.e., polymorphism of *NUDT15* codon 139, and it was shown that with thiopurine treatment, those homozygous for R139C will likely develop agranulocytosis with hair loss. In particular, the prevalence of the R139C polymorphism is high in Asia, and approximately 1 in 100 Japanese individuals is homozygous. Kakuta Y et al*.* reported in a multicenter study that *NUDT15* R139C is the best pharmacogenetic marker for predicting thiopurine-induced severe ARs, such as agranulocytosis with alopecia totalis, in Japanese patients with IBD [16]. In response to this report, *NUDT15* codon 139 analysis was included as part of insurance coverage in Japan in 2019.

The present study confirmed the relationship between *NUDT15* polymorphism(s) and leukopenia. Although *NUDT15* codon 139 analysis can predict agranulocytosis with alopecia totalis, it cannot predict all other events, such as hepatotoxicity, leukopenia and alopecia, and probably pancreatitis, events that may necessitate discontinuation of the medication. We also confirmed that *NUDT15* gene analysis can predict the occurrence of ARs; nonetheless, it should be noted that the *NUDT15* gene analysis covered by Japanese insurance only involves R139C. Seventeen of our 49 (34.7%) patients did not carry R139C but did have other polymorphisms. Therefore, even among patients with a ‘wild-type *NUDT15* gene’ based on R139C analysis via Japanese insurance practice, 34.7% harbor polymorphisms in exon 1, i.e., V18I, V18_V19insGV, of *NUDT15* and/or *ITPA* c.94c > a. Moreover, all of these patients had a significantly higher incidence of all ARs and leukopenia than patients with wild-type genes. Indeed, 7 of 39 (17.9%) patients in this study without the R139C polymorphism developed some ARs. Thus, some ARs will occur in approximately 20% of patients diagnosed as having a ‘wild-type *NUDT15* gene’ based on R139C analysis, forcing them to discontinue thiopurine use, temporarily withdraw from the treatment, or reduce their dose. Of the 5 patients who carried both *NUDT15* polymorphism and c.94c > a, 3 (60.0%) required thiopurine discontinuation, and 1 patient (20%) required temporary withdrawal. In contrast, none of the four patients with polymorphisms only in *IPTA* c.94c > a required discontinuation of thiopurine, although three required dose reduction. Patients with polymorphisms in these genes appear to be less resistant to thiopurine. It should also be noted that those who carry polymorphisms in both *ITPA* and *NUDT15* tend to develop leukopenia in a shorter period of time than those who carry only one of the polymorphisms.

A previous report revealed that homozygous *NUDT15* polymorphism has a strong relationship with alopecia totalis. In the present study, the only patient carrying a homozygous polymorphism in *NUDT15* (also had heterozygous polymorphism in *ITPA*) experienced alopecia totalis simultaneously with agranulocytosis. Thus, thiopurine administration should be absolutely avoided when a homozygous *NUDT15* polymorphism is detected. On the other hand, of the 4 patients who developed alopecia in this study, one harbored *NUDT15* polymorphism alone, one had *ITPA* polymorphism alone, one had both polymorphisms, and surprisingly, one had no polymorphisms. Therefore, it is unlikely that all drug-induced alopecia can be predicted by NUDT15 gene analysis alone.

Although the prevalence of *NUDT15* polymorphisms was significantly higher in females, it is very interesting that even greater significant differences in the incidence of ARs and leukopenia based on sex were observed. Even in cases of a wild-type genotype, the activity of TPMT varies by sex, and it has been reported that activity is low in females [18]. However, the average 6-MMP level, which appears to reflect the activity of TPMT, tended to be higher in females (360.3 ± 299.4 vs. 278.4 ± 131.1, *P* = 0.200) in this study. Hence, the sex difference with regard to the occurrence of ARs cannot be fully explained by TPMT activity.

In this study, the mean content of 6-TGNs was not related to ARs or polymorphism(s) of *NUDT15*. NUDT15 converts the thiopurine active metabolite 6-TGTP to the monophosphate thioguanosine nucleotide 6-TGMP. Polymorphisms in the *NUDT15* gene are thought to decrease enzyme activity and thus increase levels of 6-TGTP and 6-TdGTP, which are then incorporated into RNA or DNA and cause leukopenia [11]. As the 6-TGN level, which can be measured clinically, is the sum of 6-T(d)GMP, 6-T(d)GDP, and 6-T(d)GTP, it is understandable that *NUDT15* polymorphism does not affect the level of 6-TGN. Zhu X et al. reported that neither 6-TGN nor 6-MeTIMP was associated with leukopenia but that when examined separately according to the presence or absence of *NUDT15* polymorphism, a correlation between leukopenia and 6-TGN level was detected [19]. In our study, the mean 6-TGN level was calculated at 8 to 52 weeks after the start of azathioprine administration or until discontinuation due to ARs. Comparisons of subgroups were considered inappropriate in our study, as some patients discontinued medication for AR within 4 weeks.

Similar results were obtained for mean concentrations of 6-MMPs and 6-MPs. Accordingly, measurement of 6-TGNs, 6-MMPs, and 6-MPs may not be meaningful for predicting ARs due to thiopurines, as it is technically difficult to measure all metabolites that comprise these metabolites individually. Recently, it has been recommended to specifically measure the active DNA-binding triform of 6-TGN by mass spectrometry. However, the fact that this method was not adopted in our previous study in which samples were collected and analyzed from 2008 to 2011 is also considered to be one of the reasons why no correlation with the NUDT15 gene polymorphism was found.

More recently, there have been numerous reports that ITPA gene polymorphisms do not predict thiopurine toxicity. Even in studies in Asia, where the prevalence of ITPA polymorphisms is known to be high, especially the report from South India by Jena A et al. concluded that ITPA polymorphisms are not predictive of thiopurine-induced leukopenia [20]. As mentioned above, the results of this study suggest that analysis of ITPA gene polymorphism alone is insufficient for predicting thiopurine toxicity. However, carriers of both NUDT15/ITPA polymorphisms are not uncommon in Asia, and both carriers have been shown to be at additively increased risk of thiopurine toxicity. A retrospective study of 1419 Chinese patients with thiopurine-induced myelosuppression also concluded that polymorphism analysis of both the NUDT15 and ITPA genes was recommended [21].

The mechanism by which *ITPA* gene polymorphisms cause hair loss and leukopenia is not well understood. ITPase is not an enzyme that directly affects the level of 6-T(d)GTP, which inhibits nucleic acid synthesis. Nevertheless, it cannot be ruled out that the decrease in ITPase activity may affect the production of adenosine triphosphate (ATP) and guanosine triphosphate (GTP) by decreasing inosine monophosphate (IMP) in the de novo synthesis system of purine nucleotides, regardless of thiopurine use. IMP dehydrogenase inhibitors such as mycophenolate mofetil (MMF) inhibit the de novo nucleic acid synthesis pathway in which GTP is synthesized from IMP via guanosine monophosphate (GMP). MMF-induced leucopenia has also been reported [22], and it can be understood that the importance of IMP in the de novo nucleic acid synthesis pathway and the ITPA polymorphism have a significant impact on nucleic acid synthesis. One case report described a patient with microscopic polyangiitis who carried a heterozygous c.94c > a polymorphism alone and presented with agranulocytosis immediately after the dose of AZA was increased [10].

The results of this study clearly show that analysis of *NUDT15* polymorphisms is effective for predicting the onset of ARs, especially leukopenia. Although the frequency of such serious ARs requiring discontinuation of administration is not high, overconfidence in R139C analysis alone is dangerous, and great care should be taken, especially when starting thiopurine or increasing the dosage during the clinical course.

The limitations of this study are as follows: 6-MPs and 6-MMPs were measured using specimens that had been stored for a long period of time, even though they were stored in an appropriate environment. In this study, we did not specifically measure the 6-TGN triform by mass spectrometry and found no correlation with the NUDT15 polymorphism. Additionally, there was a small number of subjects of a single race, i.e., Japanese. Furthermore, no homozygous *ITPA* polymorphism carriers were included, and the risk of *ITPA* polymorphism carriers thus could not be evaluated. It is hoped that these limitations will be addressed in future prospective studies.

## Conclusion

All cases of leukopenia were associated with *ITPA* c.94c > a and/or polymorphism of *NUDT15* and the risk of developing leukopenia was synergistically increased by *ITPA* and *NUDT15* gene polymorphisms. However, there was no association between the level of azathioprine metabolites and these polymorphisms. Some ARs will occur in approximately 20% of patients diagnosed as having a ‘wild-type *NUDT15* gene’ based on R139C analysis, forcing them to discontinue thiopurine use, temporarily withdraw from the treatment, or reduce their dose and great care should be taken, especially when starting thiopurine or increasing the dosage during the clinical course.

## Data Availability

All data generated or analysed during this study are included in this published article.

## Abbreviations

XO: Xanthine oxidase
TPMT: Thiopurine *S*-methyltransferase
HGPRT: Hypoxanthine guanine phosphoribosyl transferase
ITPase: Inosine triphosphate pyrophosphatase
IMPDH: Inosine monophosphate dehydrogenase
GMPS: Guanosine monophosphate synthetase
NUDT15: Nucleoside diphosphate-linked moiety X-type motif 15
AZA: Azathioprine
6-MP: 6-Mercaptopurine
6-TUA: 6-Thiouric acid
6-MeMP: 6-Methylmercaptopurine
6-TIMP: 6-Thioinosine monophosphate
6-TIDP: 6-Thioinosine diphosphate
6-TITP: 6-Thioinosine triphosphate
6-MeTIMP: 6-Methylthionosine monophosphate
6-MeTIDP: 6-Methylthionosine diphosphate
6-MeTITP: 6-Methylthionosine triphosphate
6-TXMP: 6-Thixanthosine 5’-monophosphate
6-TGMP: 6-Thioguanosine monophosphate
6-TGDP: 6-Thioguanosine diphosphate
6-TGTP: 6-Thioguanosine triphosphate
6-MeTGMP: 6-Methylthioguanine monophosphate
6-MeTGDP: 6-Methylthioguanine diphosphate
6-MeTGTP: 6-Methylthioguanine triphosphate
6-MMPs: 6-Methylmercaptopurines
6-MPs: 6-Mercaptopurines
6-TGNs: 6-Thiguanine nucleotide
